# Cardiovascular disease in the QUEST-RA study, 10 years later

**DOI:** 10.1186/s13075-019-2025-5

**Published:** 2019-11-14

**Authors:** Antonio Naranjo, Iván Ferraz-Amaro

**Affiliations:** 1 Division of Rheumatology, Hospital Universitario de Gran Canaria Doctor Negrín, Las Palmas de Gran Canaria, Spain; 2 Division of Rheumatology, Hospital Universitario de Canarias, Tenerife, Spain

The Quantitative Patient Questionnaires in Standard Monitoring of Patients with Rheumatoid Arthritis (QUEST-RA) multicenter cohort was launched by Theodore Pincus and Tuulikki Sokka in 2005, encompassing more than 9000 patients with rheumatoid arthritis (RA) from 34 countries. In those years, there was a great interest in the comorbidities associated with RA, particularly those of a cardiovascular (CV) nature. I was especially interested in analyzing this data in the QUEST-RA database and fortunately enlisted the statistical collaboration of Miguel A. Descalzo and Loreto Carmona [1]. At that time, although methotrexate was known to reduce both mortality and CV events in RA, there remained uncertainty about the effect of TNF blockers.

In our study, hyperlipidemia and smoking were associated with a higher risk of myocardial infarction, whereas hypertension and diabetes were linked to a heightened risk of stroke. In fact, we identified an association between the presence of extra-articular manifestations of RA and an increased incidence of heart attack. The study demonstrated that the duration of exposure both to disease-modifying anti-rheumatic drugs (DMARDs) and biological agents was associated with a reduced risk of CV events [1].

In an editorial, Ronald van Vollenhoven commented that the conceptual question was whether or not the length of time a patient remains under treatment serves as an effective indicator that said patient experienced good disease control during that time [2]. The author opined that while this may not necessarily be true at the individual level, at the group level, such an approach could work reasonably well. In the author’s opinion, the study, while not definitively proving that anti-rheumatic therapy decreases the risk of CV complications, did further bolster the validity of the concept, but still needed to be formally addressed, either with a prospective cohort approach or by a controlled clinical trial [2]. Because evaluating these characteristics in a clinical trial is practically impossible, prospective cohort studies including controls could be a feasible alternative [3].

Since the publication of the QUEST-RA study, research in the area of CV comorbidity in RA, both in basic investigation and in epidemiological studies, has mushroomed. This research has included the study of future CV events through prospective studies, the presence of subclinical atherosclerosis using a wide variety of diagnostic techniques, and many other comorbidities related to CV disease in RA, including metabolic syndrome, insulin resistance, and inflammatory dyslipidemia. Moreover, the hypothesis raised by the QUEST-RA study was ultimately confirmed in the years following its publication. In this sense, several reports demonstrated that effective control of disease activity in patients with RA reduced their CV disease risk. For example, in 2015, a meta-analysis found that TNF-alpha inhibitors were associated with a reduced risk of all CV disease events, as well as of specific outcomes, including myocardial infarction and stroke [4]. Moreover, the cardiovascular benefits of TNF inhibitors were found to be limited to those RA patients whose synovitis responded to these agents [5]. For this reason, in 2016, the EULAR recommendations for cardiovascular disease risk management in patients with rheumatoid arthritis recommended that “disease activity should be controlled optimally in order to lower CV disease risk in all patients with RA” [6]. These recommendations emphasized that new evidence supported and underscored the association between higher cumulative inflammatory burden and increased CV disease risk in RA [6].

The QUEST-RA study was pioneering, not only regarding the role of biological therapies in reducing CV disease in RA, but also in the potential role of biological anti-inflammatory therapies in the prevention of CV events in patients without inflammatory diseases. In the early 2000s, the baseline plasma concentration of C-reactive protein was thought to predict the risk of future myocardial infarctions and strokes. Moreover, the reduction associated with the use of aspirin in the risk of a first myocardial infarction appeared to be directly related to the level of C-reactive protein, raising the possibility that anti-inflammatory agents may have clinical benefits in preventing CV disease [7]. Furthermore, the addition of C-reactive protein measurements to screening protocols, based on lipid levels, provided advantages in identifying persons at risk for cardiovascular events [8]. Similarly, statin therapy was found to be effective in the primary prevention of coronary events in subjects with relatively low lipid levels but with elevated C-reactive protein levels [9]. And finally, clinical trials based on biological therapies began to determine whether these therapies were capable of reducing CV events in patients without inflammatory diseases. In this sense, anti-inflammatory therapy targeting the interleukin-1β innate immunity pathway with canakinumab led to a significantly lower rate of recurrent cardiovascular events than placebo, independent of lipid-level lowering [10]. The blockade of interleukin-1 with anakinra was also shown to improve glycemia and beta-cell secretory function and reduced markers of systemic inflammation in patients with diabetes 2 [11].

In conclusion, the QUEST-RA study carried out 10 years ago has opened new research avenues in a field that has become essential to the care of patients with RA. The QUEST-RA study’s finding that treatments for RA reduced cardiovascular risk in these patients was fundamental to understanding the relationship between systemic inflammation and CV disease. This has proven to be a crucial result, not only for patients with inflammatory arthritis, but also for the field of CV disease in healthy populations.

## Data Availability

Not applicable

## Abbreviations

CV: Cardiovascular
EULAR: European League Against Rheumatism
QUEST-RA: Quantitative Patient Questionnaires in Standard Monitoring of Patients with Rheumatoid Arthritis
